# Large differences in global transcriptional regulatory programs of normal and tumor colon cells

**DOI:** 10.1186/1471-2407-14-708

**Published:** 2014-09-24

**Authors:** David Cordero, Xavier Solé, Marta Crous-Bou, Rebeca Sanz-Pamplona, Laia Paré-Brunet, Elisabet Guinó, David Olivares, Antonio Berenguer, Cristina Santos, Ramón Salazar, Sebastiano Biondo, Víctor Moreno

**Affiliations:** Unit of Biomarkers and Susceptibility, Cancer Prevention and Control Program, Catalan Institute of Oncology (ICO), Av Gran Via 199-203, E-08907 L’Hospitalet de Llobregat, Barcelona, Spain; Colorectal Cancer Group, Bellvitge Biomedical Research Institute (IDIBELL), Barcelona, Spain; Biomedical Research Centre Network for Epidemiology and Public Health (CIBERESP), Barcelona, Spain; Department of Medical Oncology, Catalan Institute of Oncology (ICO), kragujevac, Spain; Department of General and Digestive Surgery, Colorectal Unit, Bellvitge University Hospital (HUB - IDIBELL), Barcelona, Spain; Department of Clinical Sciences, School of Medicine, University of Barcelona (UB), Barcelona, Spain

**Keywords:** Colon cancer, Gene expression, Gene regulatory networks, Transcription factors, Transcriptional interactions

## Abstract

**Background:**

Dysregulation of transcriptional programs leads to cell malfunctioning and can have an impact in cancer development. Our study aims to characterize global differences between transcriptional regulatory programs of normal and tumor cells of the colon.

**Methods:**

Affymetrix Human Genome U219 expression arrays were used to assess gene expression in 100 samples of colon tumor and their paired adjacent normal mucosa. Transcriptional networks were reconstructed using ARACNe algorithm using 1,000 bootstrap replicates consolidated into a consensus network. Networks were compared regarding topology parameters and identified well-connected clusters. Functional enrichment was performed with SIGORA method. ENCODE ChIP-Seq data curated in the *hmChIP* database was used for *in silico* validation of the most prominent transcription factors.

**Results:**

The normal network contained 1,177 transcription factors, 5,466 target genes and 61,226 transcriptional interactions. A large loss of transcriptional interactions in the tumor network was observed (11,585; 81% reduction), which also contained fewer transcription factors (621; 47% reduction) and target genes (2,190; 60% reduction) than the normal network. Gene silencing was not a main determinant of this loss of regulatory activity, since the average gene expression was essentially conserved. Also, 91 transcription factors increased their connectivity in the tumor network. These genes revealed a tumor-specific emergent transcriptional regulatory program with significant functional enrichment related to colorectal cancer pathway. In addition, the analysis of clusters again identified subnetworks in the tumors enriched for cancer related pathways (immune response, Wnt signaling, DNA replication, cell adherence, apoptosis, DNA repair, among others). Also multiple metabolism pathways show differential clustering between the tumor and normal network.

**Conclusions:**

These findings will allow a better understanding of the transcriptional regulatory programs altered in colon cancer and could be an invaluable methodology to identify potential hubs with a relevant role in the field of cancer diagnosis, prognosis and therapy.

**Electronic supplementary material:**

The online version of this article (doi:10.1186/1471-2407-14-708) contains supplementary material, which is available to authorized users.

## Background

Transcriptional regulation has an essential role for proper cell functioning. Gene regulatory programs establish and maintain specific cell states [1], ensure cell homeostasis and avoid metabolic disorders [2]. Genetic regulatory information encoded in DNA binding sites, such as enhancers and promoters, is interpreted by a network of transcription factors (TFs) [3]. Epigenetic events like DNA methylation or histone modifications are regulators of transcription [4, 5] and non-coding RNAs such as siRNAs and miRNAs are also involved in gene expression regulation at the post-transcriptional level [6].

Identification of global regulatory perturbations that actively participate in the initiation and maintenance of the tumor state is one of the major challenges in cancer biology [7]. Important processes intimately related to the neoplastic process, such as development and cell differentiation, are widely mediated by gene regulation [8]. Dysregulation of signaling pathways has also been related with tumor growth and cancer progression [9]. Although specific tumor genetic alterations are well described and annotated [10], comprehensive studies are required to obtain more information about the transcriptional programs involved in tumor development. Thus, a global analysis of regulatory network perturbations still remains a fundamental challenge for cancer biology [7].

Recent bioinformatics developments make use of large-scale gene expression datasets to infer genome-wide gene regulatory networks (GRN) [11]. Although not as accurate as methods based on experimental procedures and usually requiring subsequent validation, this approach to computationally-infer regulatory networks can be useful to predict *in-vivo* functions of specific cell types [12]. Diverse methodological approaches to infer GRNs have been proposed, such as regression-based methods, correlation, information-theoretic approaches and Bayesian networks [13]. Among all those, the ARACNe algorithm for the reconstruction of GRNs has been successfully applied to reverse-engineer large-scale transcriptional networks in B-cell leukemia [14, 15], neuroblastoma [16], T cell acute lymphoblastic leukemia [17] and prostate cancer [18]. These methodologies have also been applied to analyze and compare GRNs of several human tissues [19]. However, there are a limited number of studies about gene regulatory network inference in colon cancer cells, and these analyses were restricted to a small number of genes or used small sample sizes for the inference [20–23].

The aim of our study is to infer GRNs from transcriptional data obtained for a large sample of stage II colon tumor cells and paired adjacent pathologically normal mucosa, as well as to perform a comprehensive analysis of the changes in the transcriptional regulatory programs related to the tumor phenotype.

## Methods

### Patients and samples

One hundred patients with an incident diagnosis of colon cancer who were visited at the Bellvitge University Hospital (Barcelona, Spain) between January 1996 and December 2000 were included in the study. Cases were selected to define a homogenous series of patients with stage II, microsatellite-stable, pathology confirmed adenocarcinoma of the colon. All patients underwent radical surgery and had no signs of tumor cells when margins were examined. Fresh samples were collected and frozen by the pathologist from the surgical specimen. Adjacent mucosa was obtained from the proximal margin and was at least 10 cm distant from the tumor lesion. The Clinical Research Ethics Committee (CEIC) of the Bellvitge Hospital approved the study protocol, and all individuals provided written informed consent to participate and for genetic analyses to be done on their samples. The approval number is PR178/11. Additional information about the study and patient samples can be found at http://www.colonomics.org.

### Gene expression dataset

Total RNA was isolated from tissue samples of tumor and normal adjacent mucosa using Exiqon’s miRCURY™ RNA Isolation Kit (Exiqon, Denmark), according to manufacturer’s protocol. Extracted RNA was quantified by NanoDrop® ND-1000 Spectrophotometer (Nanodrop technologies, Wilmington, DE) and stored at −80°C. RNA quality was assessed with RNA 6000 Nano Assay (Agilent Technologies, Santa Clara, CA) following manufacturer’s recommendations and was further confirmed by gel electrophoresis. RNA integrity numbers showed good quality (mean = 8.1 for tumors, and 7.5 for adjacent normal). RNA purity was measured with the ratio of absorbance at 260 nm and 280 nm (mean = 1.96, sd = 0.04), with no differences among tissue types.

RNA samples were hybridized onto the Affymetrix Human Genome U219 96-Array Plate platform (Affymetrix, Santa Clara, CA) following Affymetrix standard procedures. Annotation of the array was based on hg19 genome version. A blocked experimental design was implemented to avoid biases due to potential plate effects (i.e. all plates contained the same proportion of normal and tumor samples). After evaluating the quality of the 200 CEL files using Affymetrix standard quality parameters (e.g. level of background noise, labeling and hybridization efficiency, and RNA degradation), 4 arrays (two normal-tumor pairs) were excluded. Therefore, a final dataset of 196 arrays was used for subsequent analyses. Raw data were normalized together using the Robust Multi-array Average (RMA) algorithm [24] implemented in the *affy* package [25] of the Bioconductor suite (http://bioconductor.org). All other analyses were done with R 2.15.1 statistical computing suite (http://www.R-project.org). A model-based clustering was applied to the full expression dataset in order to detect and remove non-expressed and saturated probe-sets from further analyses.

The complete gene expression dataset was uploaded to the National Center for Biotechnology Information’s Gene Expression Omnibus Database with GEO series accession number GSE44076.

### Transcription factor selection

The list of TFs used was built by merging two different sources of information. The first one was the manually-curated compilation of human TFs reported by [26]. More specifically, 1,391 TFs classified in Supplementary Information S3 as ‘a’, ‘b’ or ‘other’ were chosen. In order to generate a broader set of putative TF genes, the collection of curated TFs was complemented with an additional set of 1,415 genes that were associated with specific GO terms related to transcription. In particular, genes associated with GO terms (GO:0045449 - Regulation of transcription, GO:0030528 - Transcription regulator activity and GO:0001071 - Nucleic acid binding transcription factor activity) were chosen. The GO database release used was 2011-03-19 accessed from AmiGO version 1.8 [27]. This yielded a set of 2,806 unique TFs, which were represented by 7,811 Affymetrix probe-sets in the expression array that was used.

### Inference, representation and analysis of transcriptional regulatory networks

Transcriptional regulatory networks were built using the ARACNe algorithm [15]. Prior to the ARACNe analysis, simulations were performed to model the optimal kernel width that allowed a proper mutual information (MI) estimation in our dataset. The null distribution of the MI was also empirically determined by simulation analysis in order to be able to further identify those significant correlations between TFs and their putative target genes. The significance p-value used as a threshold was 1e-07. ARACNe2 algorithm was run with DPI tolerance set to 0 to remove potential indirect transcriptional interactions from both networks. Remaining parameters were used with their default values. For each network, 1000 bootstrap replicates were performed and summarized to obtain more robust and accurate consensus networks. Only the giant connected component of both networks was considered for downstream analyses. Network visualization, descriptive, simple parameters estimation and figures were performed with Cytoscape software version 2.8.2 [28]. Directed graphs were used to describe networks, in which a regulatory relationship between a TF and a target gene was represented by a directed edge (i.e. arrow) between these two connected nodes, being the origin of the edge the TF. Comprehensive network topology analyses, along with the estimation of complex parameters, were carried out with the Network Analyzer Cytoscape plugin [29]. KEGG pathway enrichment analysis was performed with the SIGORA R package version 0.9.2 and default parameter values [30]. In the analysis of lost edges, a gene was considered to become silenced in the tumor if its average expression level was smaller than 4 and the log_2_ fold change between the tumor and the normal expression values was smaller than -1 (i.e. a 2-fold change decrease in the tumor). The analysis of network clusters was performed with the MINE Cytoscape plugin [31]. Only clusters with more than 10 nodes were considered for detailed analysis. Somatic mutation data were obtained from the COSMIC database [10] using the following parameters: large intestine (tissue), all (subtissue), carcinoma (histology), all (subhistology). Only genes with a mutation frequency greater than 5% were considered for further analysis.

### *In-silico*network validation

Gene annotation, (e.g. Ensembl gene id, chromosome, strand, start and end position) was retrieved through *BiomaRt* R/Bioconductor package [32]. For each gene, genomic sequence around the transcription start site +/- 1 kb according to hg18 coordinates was obtained with the *BSgenome* R/Bioconductor package version 1.24.0. The validation analysis was performed using the *hmChIP* database, which contains ChIP-Seq and ChIP-on-chip data from ENCODE experiments that represent more than 10,000,000 protein-DNA interactions [33]. Only the interactions of TFs with at least more than 20 target genes in the normal tissue network were considered for validation. For each TF, the *hmChIP* database was queried providing a list of genomic regions corresponding to the regulatory sequences of their targets in the normal tissue network. Results were rank ordered based on the degree of overlap between the uploaded genomic regions and the peak lists collected by *hmChIP* database from ChIP-Seq and ChIP-on-chip ENCODE datasets. Enrichment ratios and significance p-values for the overlaps were provided by *hmChIP* tool. Benjamini and Hochberg false discovery rates were also reported by the tool to account for multiple testing.

## Results

### Massive loss of regulatory activity in tumor cells

**Figure 1:**
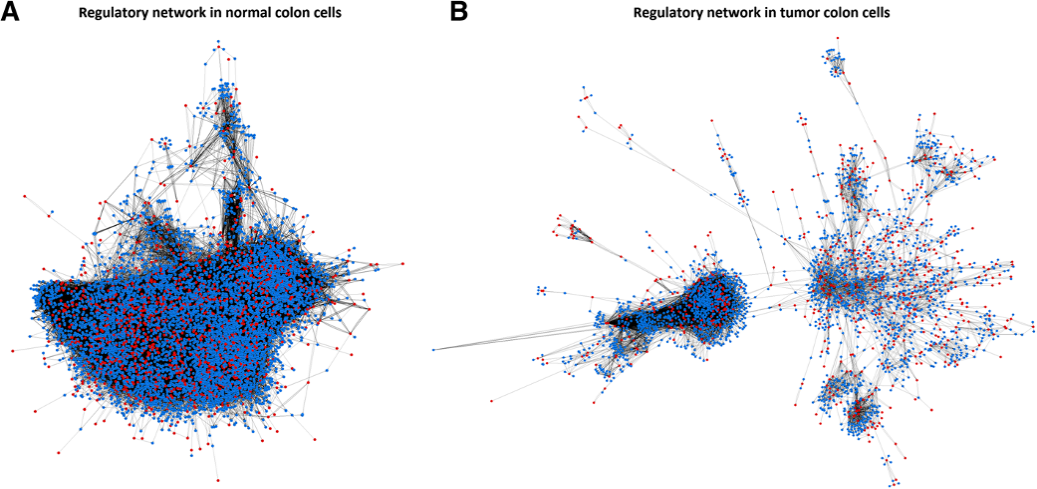
**Normal and tumor regulatory networks.** Inference and representation of normal **(A)** and tumor **(B)** regulatory networks. Both networks were inferred using microarray expression data from paired normal and tumor colon tissue obtained from the same set of individuals. Red nodes correspond to TFs and blue to non-TFs. Notice that a TF may also be the target gene of another TF. A global loss of transcriptional interactions in the tumor regulatory network is observed.

A large loss of transcriptional interactions was found in the tumor regulatory network (Figure 1, Table 1). The tumor regulatory network contained 47% fewer TFs than the network of normal cells (621 vs. 1,177), as well as 60% fewer target genes (2,190 vs. 5,466). Most nodes disappeared in the tumor network because their expression was completely unrelated to other nodes. Furthermore, the number of direct transcriptional interactions was reduced by 81% (11,585 in the tumor network vs. 61,226 in adjacent normal cells).

**Table 1:** **Networks descriptive parameters and topological features**

	Normal network	Tumor network	Ratio Tumor/Normal
***Descriptive parameters***			
Nodes	6,643	2,811	0.42
Transcription factors	1,177	621	0.53
Target genes	5,466	2,190	0.40
Edges	61,226	11,585	0.19
***Main topological features***			
Network diameter	12	17	1.42
Proportion of shortest paths	14%	4%	0.29
Characteristic path length	4.0	5.0	1.25
Average number of neighbors	16.9	7.6	0.45
Multi-edge node pairs	5,204	976	0.19

**Figure 2:**
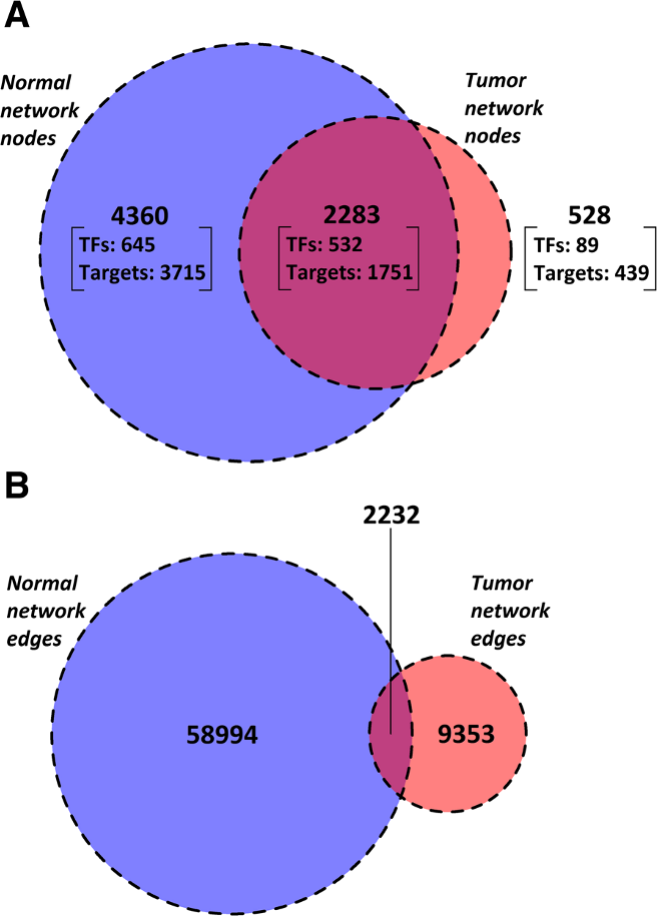
**Summary network nodes and edges overlap between normal and tumor networks.** Node **(A)** and edge **(B)** overlap between normal and tumor networks. Blue circles correspond to the normal network, red circles correspond to the tumor network, and purple areas correspond to intersections between both networks. Notice the small edge overlap between both networks (19%) even though a large part of the nodes (81%) in the tumor network are present in the normal network.

Notably, although the node overlap between both networks is large (81% of the tumor nodes are found in the normal network), only 19% of the interactions present in the tumor network are found in the normal network (Figure 2). To visualize both entire networks with Cytoscape [28] or another platform the network representations can be found online (Additional file 1). Additionally, specific TFs and their target genes (or vice versa) can explored in the project website (http://www.colonomics.org/regulatory-networks).

**Figure 3:**
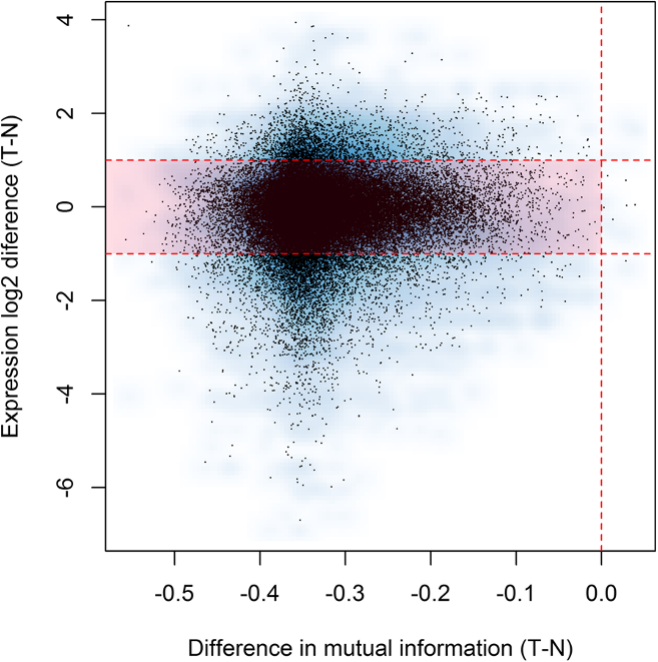
**Changes in mutual information vs. expression changes.** Each dot corresponds to a lost edge in the tumor network. X-axis represents the difference in mutual information (T-N), while the y-axis contains the expression difference between tumor and normal for either the TF or the target gene of that edge. Thus, every edge is represented by two dots in the plot. The area colored in red, where most of the dots fall, corresponds to lost interactions in the tumor (ΔMI < -0.25) in which there is no transcriptional silencing neither of the TF nor the target gene. The fact that most of the edges (~96%) fall in that region suggests that genetic or epigenetic silencing is not involved in this massive loss of transcriptional regulation in tumor cells.

**Figure 4:**
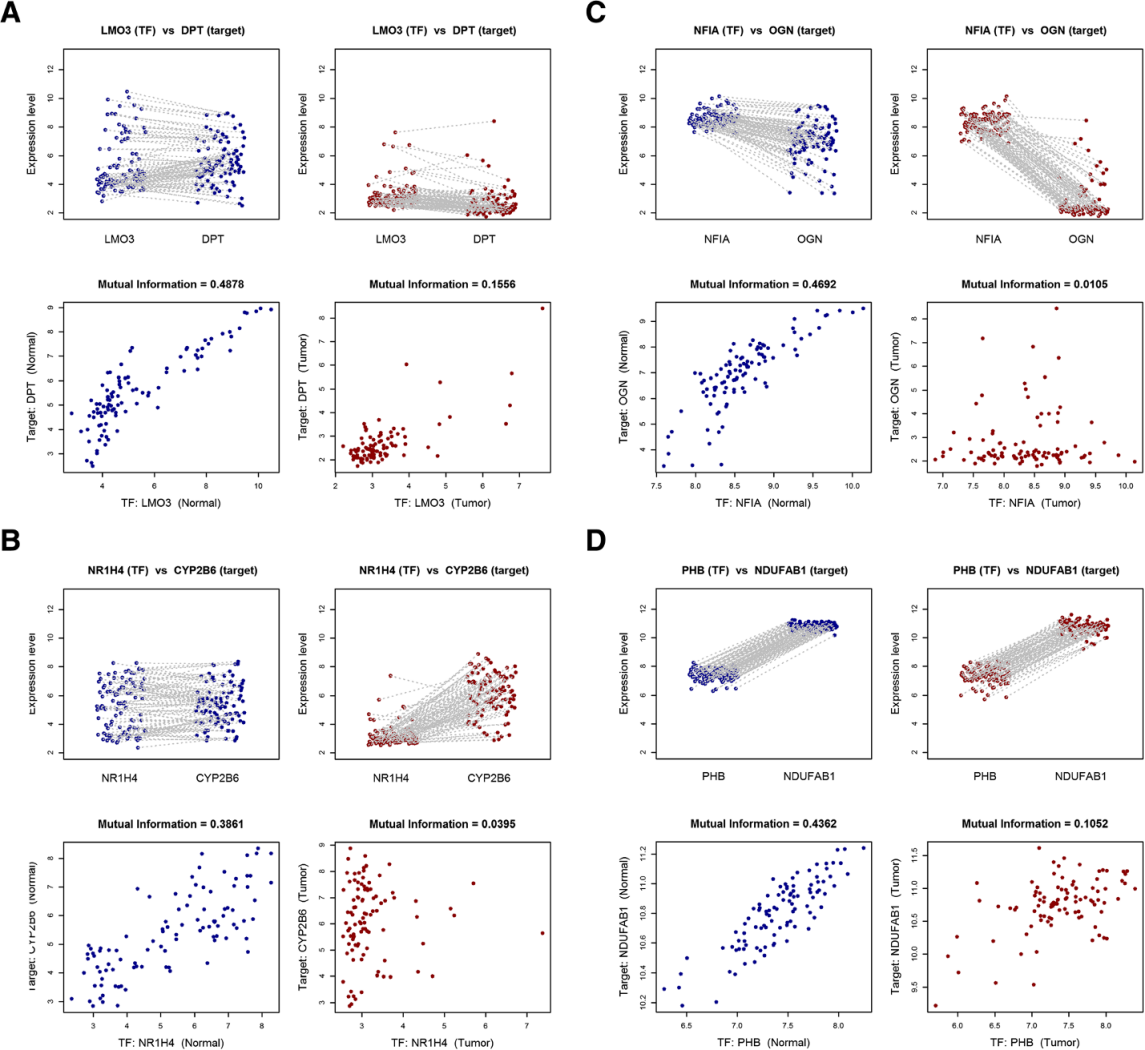
**Classification of lost edges.** The figure illustrates four examples of loss of correlation in tumor network edges. For each subfigure **(4A-4D)** the upper left plot shows the paired expression values of the TF (left) and the target gene (right) across normal samples. Similarly, the upper right plot contains the expression values across tumor samples. Lower plots show the correlation between the TF (x-axis) and the target gene (y-axis) expression for the normal samples (left) and the tumor samples (right). Blue dots correspond to expression values in normal adjacent mucosa samples and red dots correspond to expression values in tumor samples. **A)** Loss of transcriptional interaction mediated by silencing of both the TF and the target gene simultaneously. This category comprises 0.2% of lost edges (n = 80). **B)** Loss of transcriptional interaction mediated by silencing of the target gene only. This category comprises 2.1% of lost edges (n = 1,105). **C)** Loss of transcriptional interaction mediated by silencing of the TF only. This category comprises 1.7% of lost edges (n = 923). **D)** Loss of transcriptional interaction with no TF or the target gene silencing. About 96% of lost edges in the tumor network (n = 50,882) fall into this last category.

The vast majority of lost edges (76%) show a large decrease in MI but relatively small changes in gene expression (absolute log_2_ fold change < 1, Figure 3). This suggests that decreased connectivity in the tumor network was more related to transcriptional dysregulation than to gene silencing. Lost edges in the tumor network were classified into four groups according to their change in MI and gene expression change (Figure 4). Panels A-C contains examples of loss of interaction by either silencing of the TF and/or the target. These groups comprise a small proportion of lost edges (A: 80, 0.2%; B: 1,105, 2.1%; C: 923, 1.7%). Panel D shows a loss of interaction due to a decrease in the correlation, without evidence of TF or target silencing. Remarkably, most of the lost edges in the tumor network (50,882, 96%) belong to pattern D, where the loss of regulatory activity does not depend on major changes in average gene expression levels.

Loss of robustness in the tumor network was suggested by the comparison of the topological features of both networks, as shown in Table 1. Firstly, a larger distance between nodes in the tumor network was observed for different parameters, such as an increased network diameter, the characteristic path length or the decrease in average shortest paths. Secondly, a lower connectivity in the tumor network was identified according to the values of parameters related to neighborhood, such as the decrease in average number of neighbors and multi-edge node pairs. Furthermore, a characteristic of the tumor network not found on the normal was the existence of a small subset of low connected TFs with a remarkable contribution to minimal shortest paths (closeness centrality, see figure in Additional file 2). Although no significant functional enrichment was found for this set of TFs, these genes may have the potential ability to further disrupt the tumor network by breaking it into multiple disconnected components if some of their incoming our outgoing interactions are further lost. For a full set of figures of other topological features comparing the networks see Additional file 2.

### Gain of regulatory activity in tumor cells

Although the tumor network shows a large loss of transcriptional interactions, there are also specific TFs that largely increase their number of target interactions in the tumor network. A total of 91 TFs with increased activity (i.e. out-degree) and 235 up-regulated (i.e. in-degree) target genes were identified in the tumor network. The analysis of gained edges suggests a stronger role of the TFs compared to the targets. Specifically, the 91 TFs with increased activity revealed 2,224 new edges in the tumor network (24 on average, median = 12) while the 235 up-regulated targets only comprise 1,292 new transcriptional interactions (5 on average, median = 4). TFs and target genes that most increase their connectivity in the tumor network are shown in Table 2 (see complete lists in Additional file 3). KEGG pathways [34] enrichment analysis of this set of genes using the SIGORA method [30] revealed that the *Colorectal cancer* pathway (map05210) was significantly overrepresented among these TFs with increased activity (p-value = 8.9e-9). This pathway includes well-known cancer-related genes such as *FOS, TGFB3* and *TGFB1* that increased connectivity in the tumor network. In order to evaluate if this gain of regulatory activity in colon tumor cells may be related to somatic mutations we studied the degree distribution (as indicator of regulatory activity) for TFs and target genes, classified as frequently mutated (if present in COSMIC database) or not [10]. We have found that regulatory activity is independent of mutations for TFs. However, target genes included in COSMIC database showed a significant larger regulatory control than other non-mutated genes in tumors (mean in-degree 4.5 in non mutated and 7.7 in mutated, p = 0.000021). These differences were not observed in the normal network (mean in-degree 11.3 in non mutated and 12.6 in mutated, p = 0.16), indicating that mutated genes tend to loose less regulation or even increase it, since these differences were also true for targets that increased connectivity. Examples of mutated target genes that increase connectivity are *CDH11, CFH, COL3A1, COL6A3* and *COL5A2* (complete list in Additional file 4). These genes are mutated with frequency greater than 5% and show in the tumor network a large increment of regulatory activity.

**Table 2:** **Nodes with increased activity**

***TFs that most increase their activity in tumors***				
Transcription factor	Targets in Normal network	Targets in Tumor network	Gained interactions	Ratio T/N
*SNAI2*	1	119	118	119.0
*MMP14*	10	121	111	12.1
*AEBP1*	103	186	83	1.8
*BASP1*	43	123	80	2.9
*HCLS1*	91	170	79	1.9
*TFEC*	6	84	78	14.0
*DKK3*	41	112	71	2.7
*COL1A1*	62	131	69	2.1
*CD86*	74	141	67	1.9
*MAFB*	125	189	64	1.5
*NOTCH3*	18	82	64	4.6
*GLI2*	37	100	63	2.7
*TGFB1*	1	61	60	61
*GREM1*	14	70	56	5.0
*HOPX*	46	102	56	2.2
***Most up-regulated targets in Tumors***				
**Target gene**	**Targets in-degree in Normal**	**Targets in-degree in Tumor**	**Gained interactions**	**Ratio T/N**
*NNMT*	3	32	29	10.7
*CDH11*	1	24	23	24.0
*RAB31*	20	42	22	2.1
*MXRA8*	3	23	20	7.7
*RFTN1*	8	28	20	3.5
*CFH*	3	20	17	6.7
*COL3A1*	14	31	17	2.2
*EMILIN1*	12	28	16	2.3
*ENTPD1*	12	28	16	2.3
*MRC2*	7	23	16	3.3
*STAU1*	1	17	16	17.0
*AXL*	9	24	15	2.7
*OLFML2B*	10	25	15	2.5
*VCAM1*	1	15	14	15.0
*COL6A3*	12	25	13	2.1

The table lists the top 15 TFs and target genes that most increase their activity in the tumor network, sorted by the number of gained interactions. Only nodes that appeared in both networks were considered. See complete lists in Additional file 3.

### *In-silico*network validation with experimental data

Public ChIP-Seq and ChIP-on-chip datasets mainly from the ENCODE project [35] and compiled in the *hmChIP* database were used [33]. In order to avoid biases derived from tumor-specific interactions, only TFs from our normal regulatory network with available datasets from ChIP-Seq or ChIP-on-chip experiments were initially selected for validation. TFs with less than 20 targets in the normal network or showing less than 500 peaks in *hmChIP* database were filtered out to avoid focusing on tissue-specific regulations. Finally 16 TFs and their 1,443 putative target genes were selected for validation. Remarkably, though the experimental datasets were not restricted to colon tissue, 6 out of the 16 TFs (38%) showed significant overrepresentation (enrichment ratio > 1). One additional TF showed marginally significant overrepresentation in the experimental data collected in the *hmChIP* database, as shown in Table 3. This result reinforces the robustness of our inferred networks, which seem to be reasonably capturing transcriptional relationships between TFs and their target genes.

**Table 3:** ***In-silico*** **network validation**

Transcription factor (Gene Symbol)	# Targets (In normal network)	# Peaks (In hmChIP DB)	Enrichment ratio	p-value	FDR
*TCF4*	408	46,018	**1.82**	2.0e-07	3.7e-06
*NR3C1*	246	24,967	0.60	0.12	-
*PBX3*	186	39,691	0.40	0.0063	0.019
*HNF4A*	103	32,083	**2.71**	0.00027	0.0016
*TCF12*	67	54,191	**3.33**	2.0e-06	1.8e-05
*RBL2*	55	16,395	**2.33**	0.0050	0.018
*SUZ12*	50	8,742	0.62	0.12	-
*ESRRA*	42	3,284	1.50	0.37	-
*FOXP2*	42	44,482	**2.00**	0.043	0.11
*MAX*	41	16,467	1.80	0.12	-
*CDX2*	40	24,460	1.38	0.38	-
*SRF*	39	35,784	1.91	0.052	0.12
*STAT1*	35	2,804	**3.20**	0.00097	0.0044
*FOXA1*	32	21,540	0.55	0.062	0.12
*NFYB*	31	4,630	1.20	1	-
*RAD21*	26	33,302	1.40	0.50	-

Results provided by hmChIP tool containing ChIP-Seq and ChIP-chip ENCODE experiments [33]. TFs are ordered according to the number of target genes in the normal network. Cells with enrichment ratio in bold highlight significantly overrepresented TFs.

### Functional analysis of node clusters

It is known that functionally related genes tend to cluster together in network-defined biological systems (e.g. protein-protein interaction, transcriptional, or co-expression networks). Therefore, we aimed to detect clusters of genes in both the normal and tumor network to identify tumor-specific highly interconnected sub-networks, potentially enriched in relevant biological pathways. The network cluster analysis revealed 42 clusters in the normal network with more than 10 nodes. These included 953 highly interconnected genes. The tumor network included 29 clusters with 871 nodes. The distribution of nodes among clusters was similar for both networks. The list of clusters and enriched pathways (identified by SIGORA method) can be found in Additional file 5. Although most of the clusters in the tumor network were enriched in functions already present in the normal network, some clusters showed tumor-specific significant enrichments in functions with a potential role in tumor development (Table 4). More specifically, clusters 3 and 19 showed an overrepresentation of immune response pathways (e.g., Chemokine signaling pathway, Toll-like receptor signaling pathway, Cytokine-cytokine receptor interaction), and cluster 4 showed enrichment in Wnt signaling proteins. Other clusters, such as 11 and 18, also included significant enrichment of potentially relevant processes such as cell proliferation (e.g. MAPK pathway) or apoptosis, respectively.

**Table 4:** **Emergent network clusters in Tumors**

Tumor cluster*	Number of genes	Pathway	Adjusted P-value ^$^
1	120	Vascular smooth muscle contraction	1.1e-09
2	112	GnRH signaling pathway	5.9e-04
2	112	Staphylococcus aureus infection	4.8e-02
3	70	Chemokine signaling pathway	8.1e-08
3	70	Toll-like receptor signaling pathway	3.1e-07
3	70	Ether lipid metabolism	9.5e-04
4	51	Glycosphingolipid biosynthesis - ganglio series	1.6e-03
4	51	Wnt signaling pathway	1.7e-03
4	51	GnRH signaling pathway	1.3e-02
5	70	Adherens junction	1.9e-04
5	70	Chemokine signaling pathway	4.1e-02
7	44	Tight junction	5.6e-05
7	44	Tryptophan metabolism	2.4e-04
7	44	Glycosaminoglycan biosynthesis - chondroitin sulfate	4.7e-04
8	27	Adherens junction	4.0e-03
9	16	Protein digestion and absorption	4.4e-07
9	16	Adherens junction	5.9e-03
11	16	MAPK signaling pathway	2.1e-15
11	16	Prion diseases	2.4e-03
13	24	Beta-Alanine metabolism	4.4e-04
13	24	NOD-like receptor signaling pathway	9.8e-03
16	32	Glycosaminoglycan biosynthesis - chondroitin sulfate	4.5e-08
18	14	Apoptosis	2.2e-06
18	14	Nucleotide excision repair	1.0e-03
19	14	Cytokine-cytokine receptor interaction	1.4e-02
21	13	Butanoate metabolism	5.6e-05
21	13	Amino sugar and nucleotide sugar metabolism	3.4e-03
22	12	Glutathione metabolism	3.4e-04
23	18	DNA replication	6.7e-06
25	32	Vascular smooth muscle contraction	3.7e-06
28	12	DNA replication	9.6e-05

*Only clusters with significant enriched functions in tumors not already present in normal are shown.

^$^P-value for functional enrichment derived from SIGORA method.

## Discussion

In this study we have reverse-engineered the transcriptional regulatory networks of both pathologically normal and tumor colon cells obtained from the same set of patients. Using a large-scale gene expression microarray dataset, the ARACNe algorithm was applied to both tissue types independently. ARACNe gives preference to identify direct transcriptional regulatory interactions between TFs and their target genes. When both networks are compared, the most outstanding feature is the considerable loss of transcriptional interactions found in tumor cells (81%), with a global significant decrease in TFs (47%), target genes (60%). The fact that both normal and tumor samples belong to the same set of individuals, as well as the carefully performed experimental design to prevent biases between tissue types, strongly suggests that these large differences between networks are mainly due to the tumor phenotype.

Most of the TFs and target genes involved in disrupted interactions in the tumor network still maintain their expression levels, while only a minor proportion of lost edges may be explained by a complete loss of expression of one or both interactors. This expression silencing may be attributed either to genomic (e.g. DNA deletions, somatic mutations in promoter regions that hinder TF binding, transcript-truncating alterations, etc.) or epigenomic mechanisms (e.g. miRNA-associated transcript degradation, promoter hypermethylation, alterations in chromatin activation and repression marks, etc). On the other hand, disrupted interactions involving TFs and target genes that maintain expression levels in normal and tumor cells may be attributed to multiple reasons: presence or absence of a third-party molecule that could be acting as a post-translational modulator of the TF activity (i.e. phosphorylation, acetylation, ubiquitination) [36], alteration of key co-factors [1], or alterations in promoter regions that could create new TF-binding sites in target genes [37, 38]. The small set of genes involved in the loss of interactions through TFs or target gene silencing (~4%) is more likely to belong to currently known altered colon cancer pathways as the Wnt signaling and others, due to apparent under-expression. However, the vast majority of lost edges would not be easy to identify just by exploring the expression values of their TFs or targets genes. We think new and interesting undescribed mechanisms for molecular biology of colon cancer might be related to this gene deregulation without average gene expression change. A potential limitation may be the tumor cellular heterogeneity that could also be contributing to the observed loss of connectivity. While normal mucosa is a relatively homogeneous tissue among subjects, tumors are more heterogeneous, with diverse predominant cellular clones (epithelial, stromal and derived from the immune system). This could result in an apparent global loss of correlation if diverse transcriptional networks were mixed in the tumor.

The network of tumor cells also showed the emergence of a new set of transcriptional interactions that may have an essential role in tumor development and the acquisition of new cellular abilities. Recent studies have demonstrated that the activation of a small regulatory module is necessary and sufficient to initiate and maintain an aberrant phenotypic state in brain tumors [16]. Therefore, network inference approaches could prove effectively useful to uncover new modules and the master regulators that orchestrate malignant transformation. Among the TFs ranked at the top of the list of increased connectivity, our analysis identified colorectal cancer related genes: two oncogenes (*MAFB* [39] and *GLI2* [40]), proliferation-related genes (*NOTCH3* [41] and *TGFB1* [42])*,* epithelial-mesenchymal transition (*SNAI2* [43]) and the Wnt signaling genes *SFRP4*, *TWIST1*, *SMARCA4* and *DKK3*, potentially involved in colorectal cancer angiogenesis [44]. One remarkable gene with increased activity in the tumor network was *GREM1*. This gene encodes a member of the bone morphogenic protein antagonist family and may play a role in regulating organogenesis, body patterning and tissue differentiation. Interestingly, *GREM1* has been previously related with a locus strongly associated with increased colorectal cancer risk [45]. Moreover, increased expression of *GREM1* has also been recently found in colorectal polyps [46], as well as in the dysplasia to carcinoma transition in colon tumors [47]. Therefore our results suggest that *GREM1* may be mediating its tumorigenic effect by the activation of a large transcriptional program. Furthermore, encouraging results were obtained in the study of the relationship of somatic mutations in colorectal tumors in the set of relevant genes identified through our network approach. Though frequent mutation was independent of regulatory activity for TFs, we observed an association for target genes, with larger regulatory activity among mutated genes. Though this was a correlation analysis using external data from COSMIC database (we do not know if our tumors were actually mutated), it is suggestive that mutated genes trigger a regulatory control in the tumor. The presence of mutations combined with the alteration in their transcriptional regulatory connectivity postulate these genes as strong candidates to be involved in the pathogenesis of colon cancer, and even other type of tumors.

The analysis of network clusters has identified relevant sub-networks of highly connected genes specific of tumors. The regulatory network of normal cells is large and compact. Only 42 clusters have been identified with more than 10 genes. These clusters only account for 14% of the network genes, indicating that there is extensive regulation, but relatively low modularity. The tumor cell, however, has revealed 29 clusters that include 30% of their genes. This is consistent with a more modular organization of the regulatory machinery, which is also evident from the network representation (Figure 1). The functional analysis of these clusters has shown significant enrichment of known tumor-specific pathways: immune response, Wnt signaling, DNA replication, cell adherence, apoptosis, DNA repair, among others (Table 4). Some specific metabolism pathways appear also specifically captured by this analysis of sub-networks, which may be candidate for intervention: glycosphingolipid biosynthesis, tryptophan metabolism, glycosaminoglycan biosynthesis (chondroitin sulfate), beta-alanine metabolism, butanoate metabolism, glutathione metabolism. Obviously, all these functions are present in the normal cell, but they seem enhanced at the transcriptional level in the tumor, in such a way that a large cluster of related genes appear as a relevant entity. In this analysis we have generally focused on the gain of activity in the tumor network rather than on the lost interactions, given the massive loss of tumor network interactions that difficult to detect enriched functions. Despite this intrinsic limitation, we want to emphasize that the transcriptional loss found may influence the emergence of new functionality in the tumor cells. This finding may have a potential impact on the future of cancer molecular biology at level of further experiments and their corresponding biological interpretations.

The inference of GRNs has already been successfully applied to other malignances such as leukemia [14], breast cancer [48, 49] or ovarian tumors [50], with relevant findings regarding breast cancer metastasis prognostic markers or prioritization of druggable gene targets for ovarian cancer. In colorectal cancer some researchers have also explored the reconstruction of GRNs, but with limited approaches to one transcription factor [23] or only tumor tissue [21, 22]. To our knowledge, this is the first study in colon cancer that has simultaneously inferred networks for both tumor and adjacent normal cells obtained from the same set of individuals with a consistent methodology that makes both networks totally comparable.

We are aware that computational approaches of network reverse-engineering may suffer from intrinsic limitations. Therefore, we attempted a validation of the network to reinforce the validity of our study. An initial attempt to *in-silico* identify expected TF binding sites in targets was rejected because of the limited number and relative quality of the available TF positional weight matrices both in JASPAR [51] and TRANSFAC Public [52] databases. Other approach to validate the inferred regulatory networks would be to replicate our results in another colon cancer dataset. This has not been possible due to the lack of proper datasets to replicate the findings. The ARACNe’s authors emphasize in their papers that a hundred samples is the minimum sample size required to infer transcriptional networks with proper accuracy and they specifically discourage users to apply their algorithm on small datasets [15, 53]. The TCGA project [54] only provides 23 normal-tumor colon pairs available and we were unable to find a dataset with a more than 50 samples available after an exhaustive search in the most comprehensive public gene expression databases (GEO and ArrayExpress). Over the last decade, ChIP-on-chip and especially ChIP-Seq assays have become gold standard techniques for large-scale protein-DNA interaction identification. Therefore, ChIP-Seq and ChIP-on-chip datasets from the ENCODE project were used to validate interactions inferred by ARACNe. Since we restricted the potential set of TFs to be validated to those that had more than 20 interactions in the normal network and more than 500 experimentally observed peaks, only a very small part of the network could be tested. However, the obtained results were encouraging since 6 of the 16 tested TFs showed a good level of agreement. The large differences between the number of experimentally detected peaks and the number of inferred target genes for each one of the TFs may suggest a high rate of false negative interactions in our inferred networks, though it is not easy to interpret ChiP data, that provides may peaks that are not necessarily related to direct transcription interactions [55]. Failure in the validation of some TFs might also be partially influenced by the failure of the algorithm to completely remove indirect associations from the network due to high order interactions. In this direction, an extension of the ARACNe algorithm (hARACNe) specifically designed to deal with n-order interactions has been recently released, showing a significant increase in the quality and robustness of the inferred network [56]. Network deconvolution solutions over correlation-based networks have also proven to be successful for this purpose [57]. Due that the large heterogeneity of cell line tissues explored in the ENCODE project, we positively consider the overall observed level of agreement (38%), which is in the same range as previous studies found for other inferred transcriptional networks [14].

## Conclusion

The inference of direct transcriptional networks at the whole-genome level has allowed us to detect a predominant loss of transcriptional activity in colon tumor cells, which has not been described before to the best of our knowledge. However, some specific TFs and biological processes related to colon cancer also increased the connectivity and became hubs in the dysregulated tumor network. These findings will allow a better comprehension of the transcriptional regulatory programs altered in colon cancer and could be an invaluable methodology to identify potential hubs with a relevant role in the field of cancer diagnosis, prognosis and therapy.

## Electronic supplementary material

Additional file 1: **The two networks representation.** (ZIP 2 MB)

Additional file 2: **Complex networks parameters.** (PDF 1 MB)

Additional file 3: **Full list of nodes that increase their activity.** (XLS 59 KB)

Additional file 4: **Genes with altered activity and mutations in COSMIC database.** (XLS 42 KB)

Additional file 5: **Clusters enrichment analysis.** (XLS 67 KB)
