# Supplementary material for: Large differences in global transcriptional regulatory programs of normal and tumor colon cells
Source: BMC Cancer. 2014 Sep 24;14:708. doi: 10.1186/1471-2407-14-708 (PMC4182786; doi:10.1186/1471-2407-14-708)
Supplement: Supplementary file 2 — Additional file 2: Complex networks parameters. (PDF 1 MB) [file 12885_2014_4884_MOESM2_ESM.pdf]

Supporting Information 2: *Complex networks parameters*

2. A. Closeness centrality

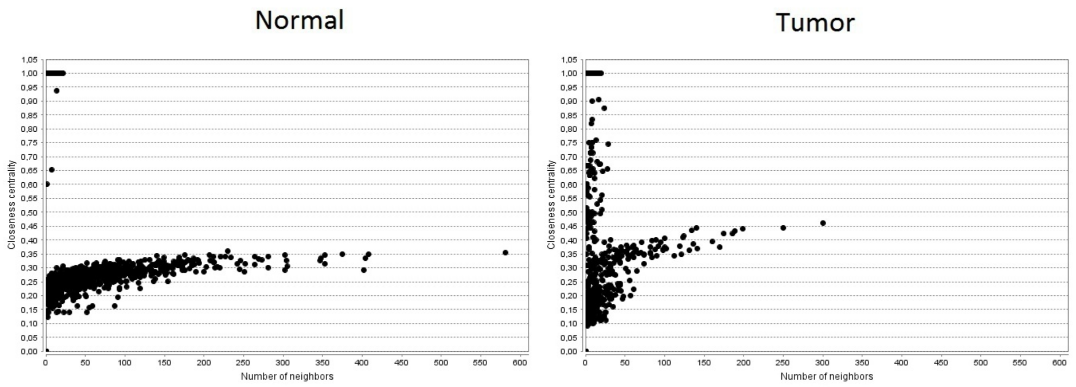

Closeness centrality in both normal and tumor networks nodes against the number of neighbors. The closeness centrality of a node is defined as the reciprocal of the average shortest path length and is a measure of how fast information spreads from a given node to other reachable nodes in the network. The closeness centrality of each node is a number between 0 and 1 and for the isolated nodes is equal to 0.

2. B. Clustering coefficients

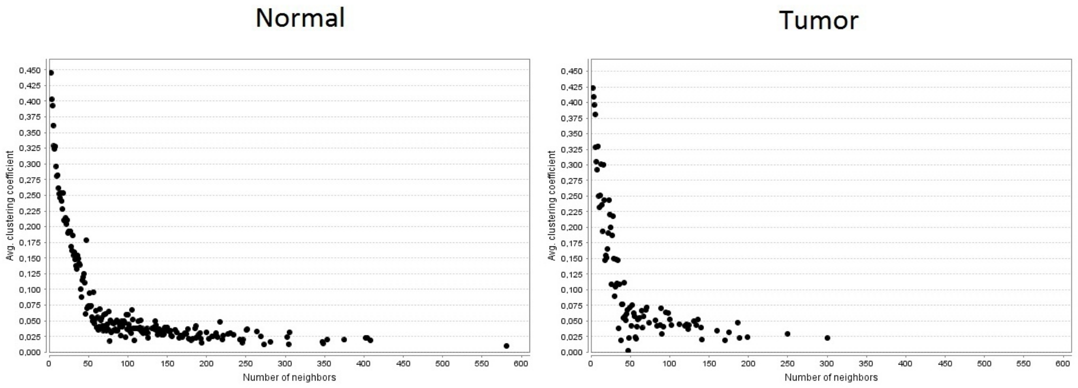

Average clustering coefficient in both normal and tumor networks nodes against the number of neighbors. The clustering coefficient of a node is a ratio N / M, where N is the number of edges between the neighbors of the node, and M is the maximum number of edges that could possibly exist between the neighbors of the node. The clustering coefficient of a node is always a number between 0 and 1. The average clustering coefficient distribution gives the average of the clustering coefficients for all network nodes with k neighbors.

2. C. Betweenness centrality

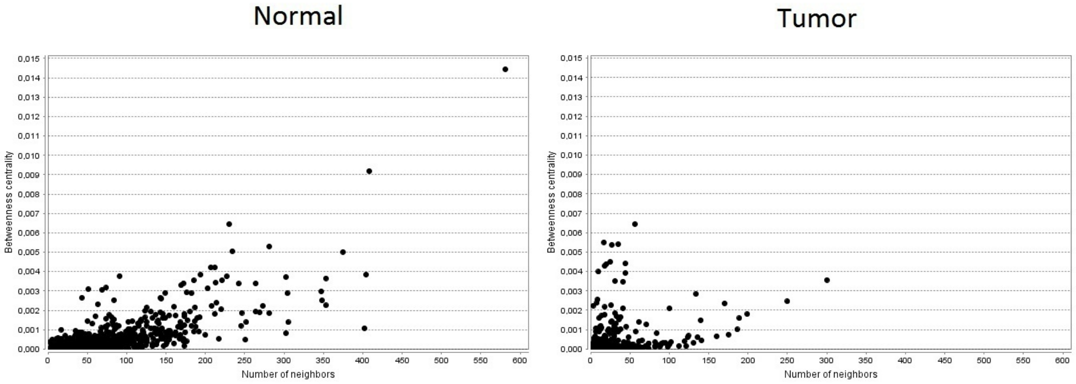

Betweenness centrality in both normal and tumor networks nodes against the number of neighbors. The betweenness centrality of a node reflects the amount of control that this node exerts over the interactions of other nodes in the network. This measure favors nodes that join communities (dense subnetworks), rather than nodes that lie inside a community. The betweenness centrality of each node is a number between 0 and 1.

2. D. Neighborhood connectivity (in and out)

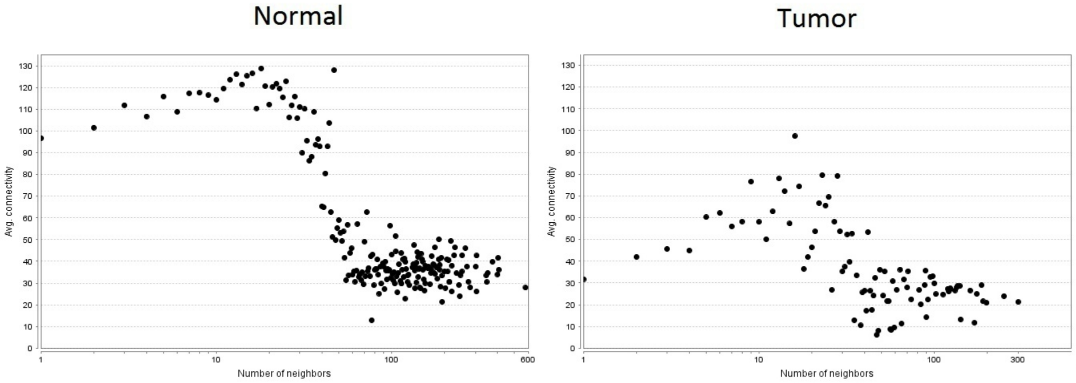

Average neighborhood connectivity (in-degree and out-degree) in both normal and tumor networks nodes against the number of neighbors. The figures show the average connectivity of all neighbors of a node, ignoring the edge direction (treating the network as undirected). The connectivity of a node is the number of its neighbors. The neighborhood connectivity of a node is defined as the average connectivity of all neighbors of the node. The neighborhood connectivity distribution gives the average of the neighborhood connectivities of all nodes with k neighbors.

2. E. Neighborhood connectivity (only out)

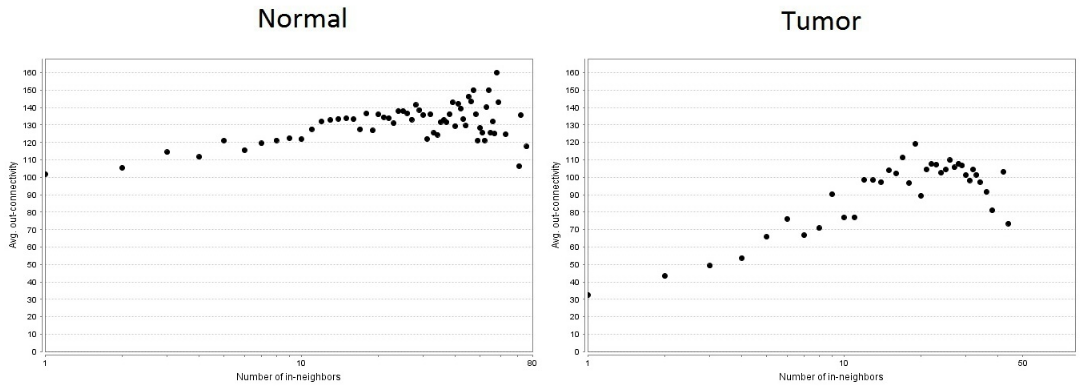

Average neighborhood connectivity (only out-degree) in both normal and tumor networks nodes against the number of neighbors. The figures show the average out-connectivity of all out-neighbors of a node (treating the network as directed). The connectivity of a node is the number of its neighbors. The neighborhood connectivity of a node is defined as the average connectivity of all neighbors of the node. The neighborhood connectivity distribution gives the average of the neighborhood connectivities of all nodes with k neighbors.

2. F. Neighborhood connectivity (only in)

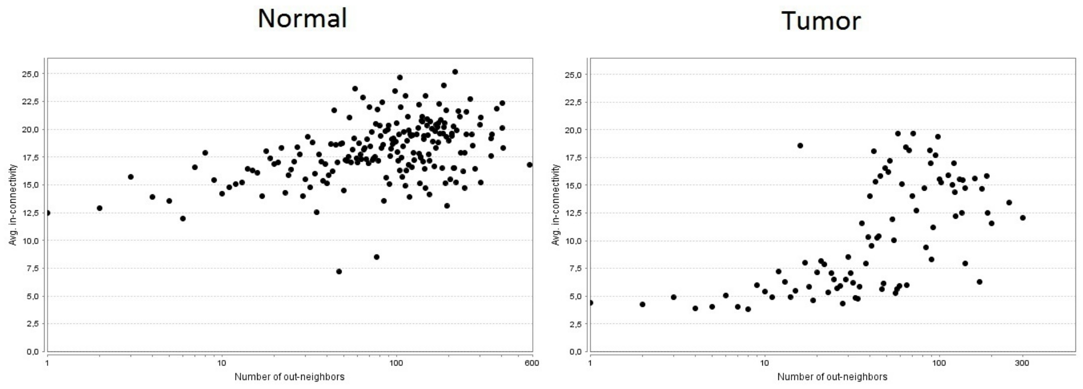

Average neighborhood connectivity (only in-degree) in both normal and tumor networks nodes against the number of neighbors. The figures show the average in-connectivity of all in-neighbors of a node (treating the network as directed). The connectivity of a node is the number of its neighbors. The neighborhood connectivity of a node is defined as the average connectivity of all neighbors of the node. The neighborhood connectivity distribution gives the average of the neighborhood connectivities of all nodes with k neighbors.

2. G. In-degree distribution

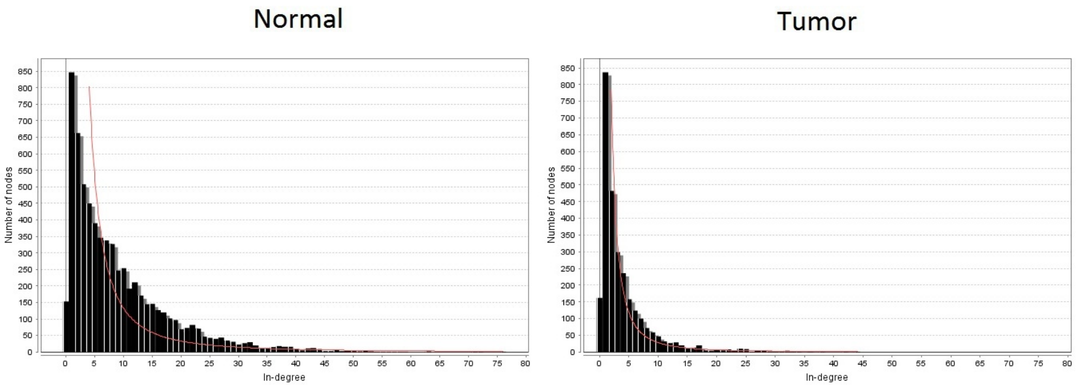

Histogram showing the in-degree frequencies in both normal and tumor networks against the number of nodes with k degrees. In undirected networks, the node degree of a node is the number of edges linked to that node. A self-loop of a node is counted like two edges for the node degree. Similarly, in directed networks the in-degree of a node is the number of incoming edges. The degree distribution can be used to distinguish between random and scale-free network topologies.

2. H. Out-degree distribution

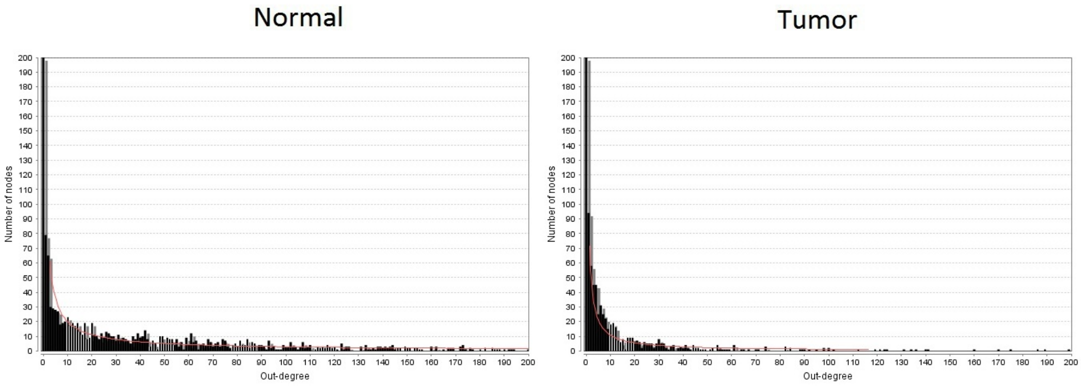

Histogram showing the out-degree frequencies in both normal and tumor networks against the number of nodes with k degrees. In undirected networks, the node degree of a node is the number of edges linked to that node. A self-loop of a node is counted like two edges for the node degree. Similarly, in directed networks the out-degree of a node is the number of outgoing edges. The degree distribution can be used to distinguish between random and scale-free network topologies.

2. I. Shortest paths distribution

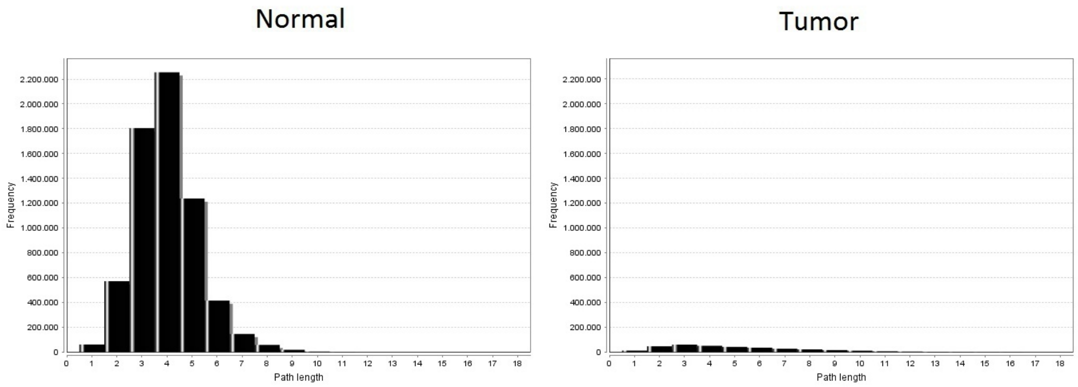

Histogram showing the shortest path length distribution in both normal and tumor networks against the number of node pairs with a given length between them. The shortest path length distribution and network diameter may indicate small-world properties of the analyzed network. The network diameter is the maximum length of shortest paths between two nodes. If a network is disconnected, its diameter is the maximum of all diameters of its connected components.

2. J. Stress centrality

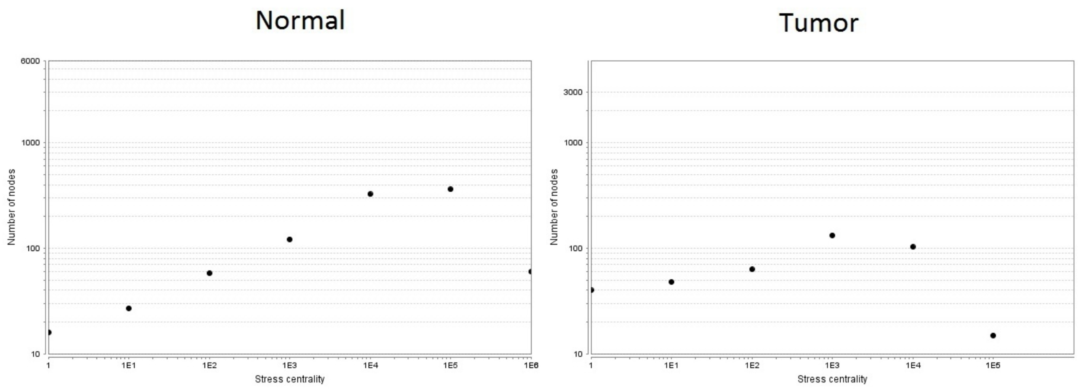

Number of nodes in both normal and tumor networks against the stress centrality. The stress centrality of a node is the number of shortest paths that pass through it. A node has a high stress if it is traversed by a high number of shortest paths. This parameter is defined only for networks without multiple edges. The stress centrality distribution gives the number of nodes with stress s for different values of s. The values for the stress are grouped into bins whose size grows exponentially by a factor of 10. The bins used for this distribution are {0}; [1, 10); [10, 100); ...

2.K. Mutual Information Distribution for the edges of the 91 TF with increased connectivity in the tumor network

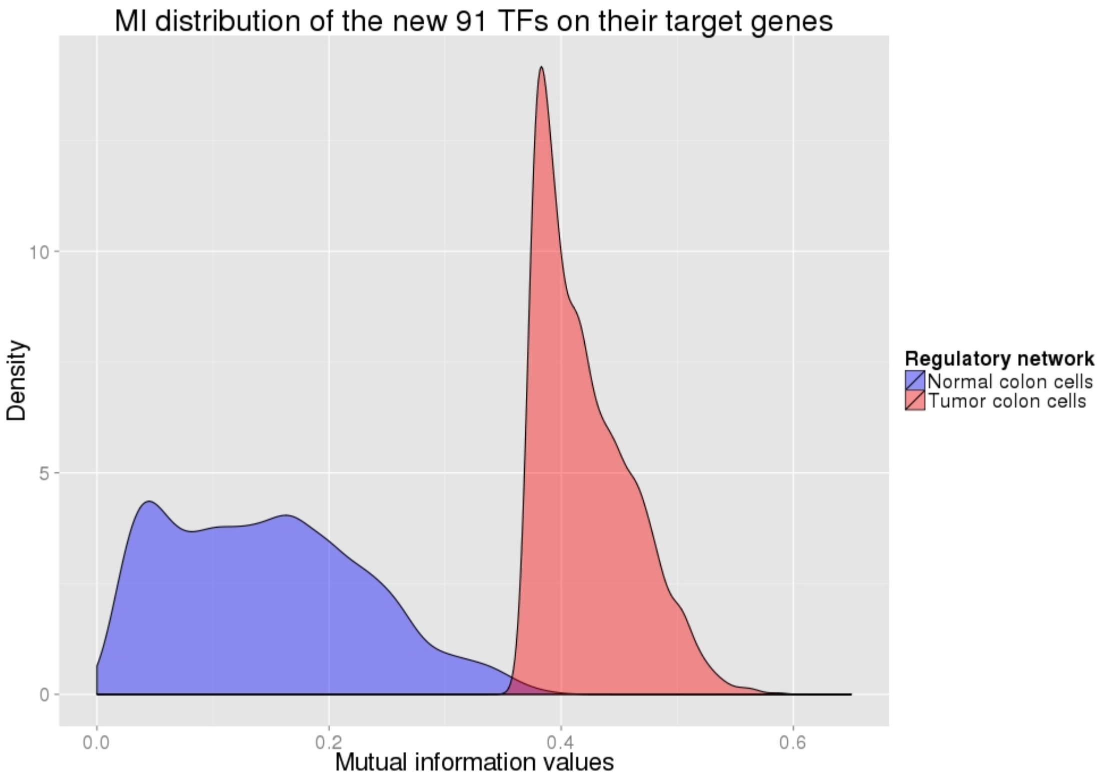

Distribution of the mutual information (MI) values for the 2,224 transcriptional interactions that are present in the tumor network but are missing in the normal network. As it is expected, the MI for the interaction in the tumor network is much higher than the interactions in the normal network.
